# Triple immune impairment in an elderly patient with IgG4-AIP and MGUS complicated by dual severe infections over 3 years: A case report

**DOI:** 10.1097/MD.0000000000051536

**Published:** 2026-11-06

**Authors:** Wanman Li, Jiyuan Liang, Yao Xie, Tingting You, Li Chen, Jinhu Wei

**Affiliations:** a Department of Clinical Laboratory, Liuzhou Municipal Liutie Central Hospital, Liuzhou, Guangxi Province, China; b Department of Neurology, The Second Affiliated Hospital of Guangxi University of Science and Technology, Liuzhou, Guangxi Province, China; c Department of Hematology, Liuzhou Municipal Liutie Central Hospital, Liuzhou, Guangxi Province, China.

**Keywords:** case report, dual opportunistic infection, IgG4-related autoimmune pancreatitis, monoclonal gammopathy of undetermined significance, triple immune impairment

## Abstract

**Rationale:**

Reports of IgG4-related autoimmune pancreatitis (IgG4-AIP) combined with monoclonal gammopathy of undetermined significance (MGUS) are mostly limited to short-term follow-up and single infectious complications. To date, no published literature has described octogenarian patients with this overlap complicated by 2 severe concurrent opportunistic infections together with 3-year serial immune-biomarker monitoring. Long-term follow-up data regarding multiple opportunistic infections under continuous immunosuppressive therapy remain scarce for this rare clinical entity.

**Patient concerns:**

An 82-year-old male patient was diagnosed with IgG4-AIP superimposed by IgGλ-type MGUS. Markedly elevated serum IgG4 (3278.00 μg/mL) and a monoclonal paraprotein level of 2.34 g/L were noted at baseline. Initial combined glucocorticoid-based immunosuppressive therapy partially improved pancreatic lesions. Subsequently, he developed concurrent *Staphylococcus hominis* bacteremia and active pulmonary tuberculosis.

**Diagnoses:**

Three-tier superimposed immune impairment, consisting of chronic IgG4-related inflammation, MGUS-associated clonal plasma-cell dysregulation, and drug-induced lymphopenia, was considered the key driver for the dual life-threatening opportunistic infections.

**Interventions:**

Standardized antibacterial and antituberculosis therapeutic regimens were administered to treat the dual infections. Immunosuppressive strategies were adjusted for this high-risk geriatric patient.

**Outcomes:**

Complete resolution of infectious lesions was achieved. Over the 36-month longitudinal surveillance period, serum paraprotein remained stable without progression to multiple myeloma, while serum IgG4 concentrations fluctuated independently of MGUS activity. Serial immune profiling confirmed persistent cellular immunodeficiency in this elderly MGUS carrier. This constitutes the first reported octogenarian patient with overlapping IgG4‑AIP and IgGλ‑MGUS suffering from dual severe opportunistic infections following immunosuppressive therapy.

**Lessons:**

Persistent cellular immunodeficiency substantially elevates the risk of life-threatening opportunistic infections in elderly patients with concomitant IgG4-AIP and MGUS. Cyclophosphamide should be used with extreme caution in this vulnerable geriatric subgroup. Pathogenetic mechanisms, infectious risk factors, and preventive considerations for this rare overlapping immune disorder deserve clinical attention.

## 1. Introduction

IgG4-related disease (IgG4-RD) is a chronic fibroinflammatory disorder with type 1 autoimmune pancreatitis (IgG4-AIP) as its classic pancreatic manifestation.^[1]^ Monoclonal gammopathy of undetermined significance (MGUS) is a premalignant plasma cell dyscrasia, and their coexistence is rare in clinical practice. Existing studies mainly focus on early diagnosis and short-term treatment, lacking long-term follow-up data on immunosuppression-related infectious complications.^[2–4]^

Chronic IgG4-RD inflammation drives clonal plasma cell proliferation, suggesting shared pathogenic pathways with MGUS.^[5]^ Combined glucocorticoid and immunosuppressant therapy effectively controls IgG4-AIP but suppresses host immunity. For elderly patients with both diseases, superimposed immune defects markedly increase the risk of fatal opportunistic infections.^[6]^ No unified guidelines are available for infection risk stratification, prophylaxis, and personalized treatment in this special population.

A short preliminary report describing this patient’s initial diagnosis at age 79 has been previously published without follow‑up data.^[4]^ Complete 3-year longitudinal data (patient aged 79–82) are presented, focusing on 2 concurrent severe infections: *Staphylococcus hominis* bacteremia and active pulmonary tuberculosis, together with serial lymphocyte and cytokine monitoring. Based on these 36-month clinical observations, a triple immune impairment hypothesis and a 3-tier management algorithm are proposed for similar overlapping immune disorders. This case report was reviewed and approved by the Ethics Committee of Liuzhou Municipal Liutie Central Hospital (Ethics approval number: KY2026‑003‑01). The study was conducted in accordance with the principles of the Declaration of Helsinki. Written informed consent was obtained from the patient for publication of clinical data, laboratory findings, and all related imaging images.

## 2. Case presentation

This 82-year-old male was admitted in June 2023 due to recurrent bilateral pleural effusions and refractory epigastric pain lasting 1 week. He had a prior history of percutaneous coronary intervention (PCI) for coronary stenosis. Physical examination revealed unintended weight loss and intermittent cough; fever, hemoptysis, drug allergy, and clear pathogen exposure were absent.

Core laboratory and imaging findings supporting dual diagnosis are summarized below.^[4]^ Bone marrow cytology showed 4.0% morphologically normal plasma cells consistent with MGUS criteria (Fig. 1B), and immunofixation electrophoresis detected a 2.34 g/L IgGλ paraprotein peak (Fig. 1A). Serum testing presented markedly elevated IgG4 (3278.00 μg/mL) plus hypocomplementemia (C3: 0.6 g/L and C4: 0.05 g/L), with positive antinuclear antibody and mildly raised anti-Sjögren’s syndrome A antibody without Sjögren’s syndrome manifestations. Urinary free light chains showed mild abnormalities, while antineutrophil cytoplasmic antibody screening excluded systemic vasculitis. Microbiological cultures of respiratory and pleural samples were negative for acid-fast bacilli and common pathogens. Abdominal axial computed tomography (CT) revealed typical diffuse pancreatic enlargement with blurred peripancreatic fat borders, characteristic of type 1 AIP (Fig. 1C). Chest CT only displayed mild bilateral inflammatory infiltrates and minor effusions without tuberculous lesions, matching his prior PCI vascular calcification findings.

**Figure 1.**
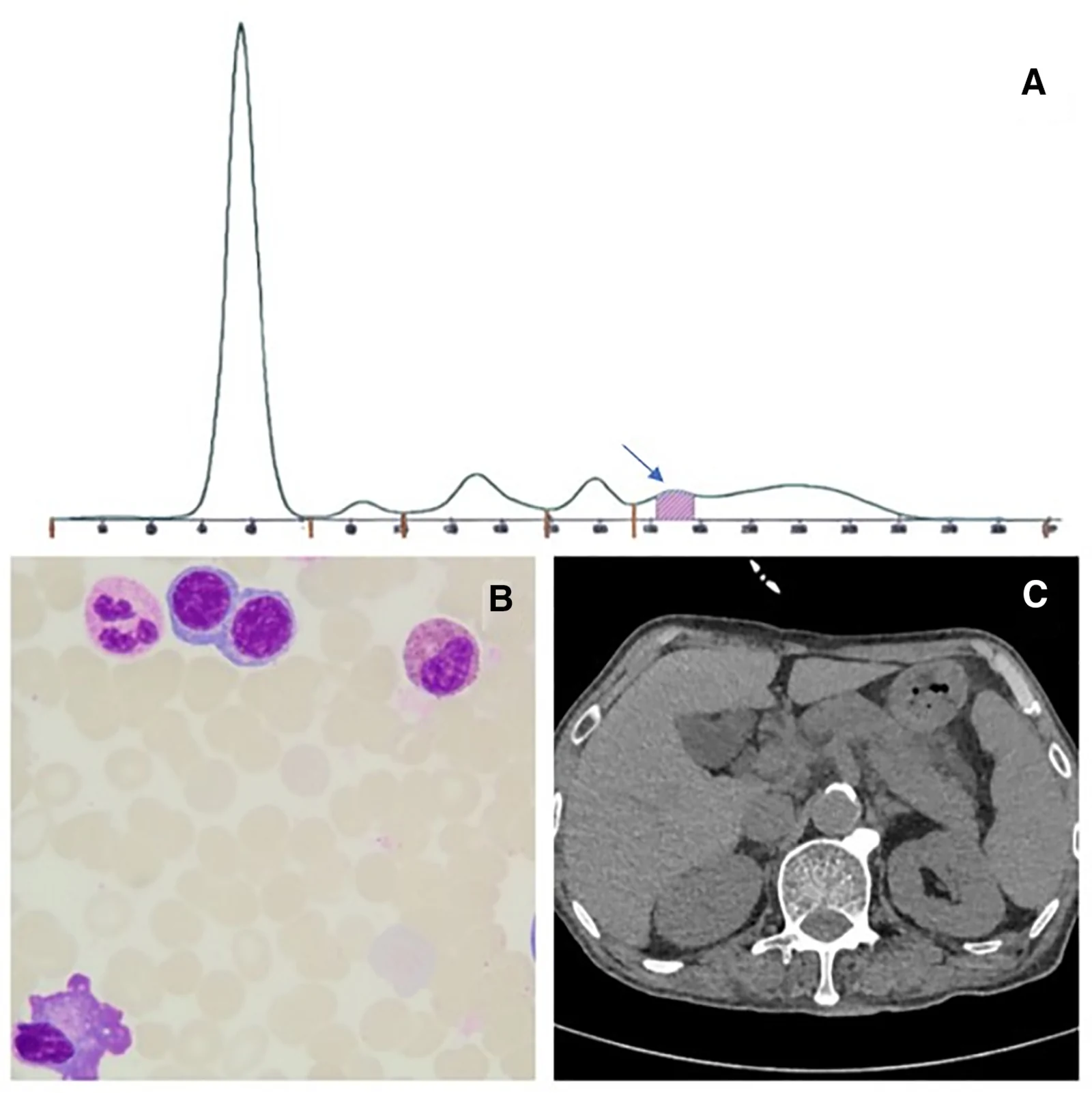
(A) SPEP shows a 2.34 g/L IgGλ paraprotein peak (arrow). (B) Bone marrow smear with 4.0% plasma cells, confirming MGUS. (C) CT showing diffuse pancreatic enlargement typical of IgG4-AIP. CT = computed tomography, IgG4-AIP = IgG4-related autoimmune pancreatitis, MGUS = monoclonal gammopathy of undetermined significance, SPEP = serum protein electrophoresis.

The patient reached 21 points (cutoff ≥ 20) under the 2019 American College of Rheumatology/European Alliance of Associations for Rheumatology IgG4-RD scoring system,^[7]^ satisfying diagnostic criteria for concurrent IgG4-AIP and IgGλ-MGUS.^[2,3,8]^

### 2.1. Initial immunosuppressive intervention

For early disease control, daily 40 mg intravenous methylprednisolone combined with twice-daily 0.75 g oral mycophenolate mofetil was initiated. Subsequent hepatic adverse effects necessitated withdrawal of mycophenolate mofetil, and the regimen was switched to oral cyclophosphamide 50 mg once daily. Two weeks after treatment initiation, epigastric pain relieved, serum IgG declined, and follow-up CT demonstrated alleviated pancreatic swelling and reduced pleural effusion.

In May 2024, the patient was readmitted with persistent high fever peaking at 38.5°C, cough, and progressive fatigue. Laboratory tests showed leukocytosis, drastically elevated high-sensitivity C-reactive protein (hs-CRP) (162.99 mg/L), and erythrocyte sedimentation rate (120 mm/h). Blood culture confirmed *S. hominis* bacteremia. Chest CT showed patchy pulmonary infiltrates plus an apical right upper lobe cavity with tiny bilateral pleural effusions, highly suggestive of secondary tuberculosis (Fig. 2A). Bronchoalveolar lavage acid-fast smear, tuberculosis nucleic acid test, and interferon-gamma release assay (IGRA) all returned positive, and the isolate was rifampicin-sensitive, confirming concurrent active tuberculosis.

**Figure 2.**
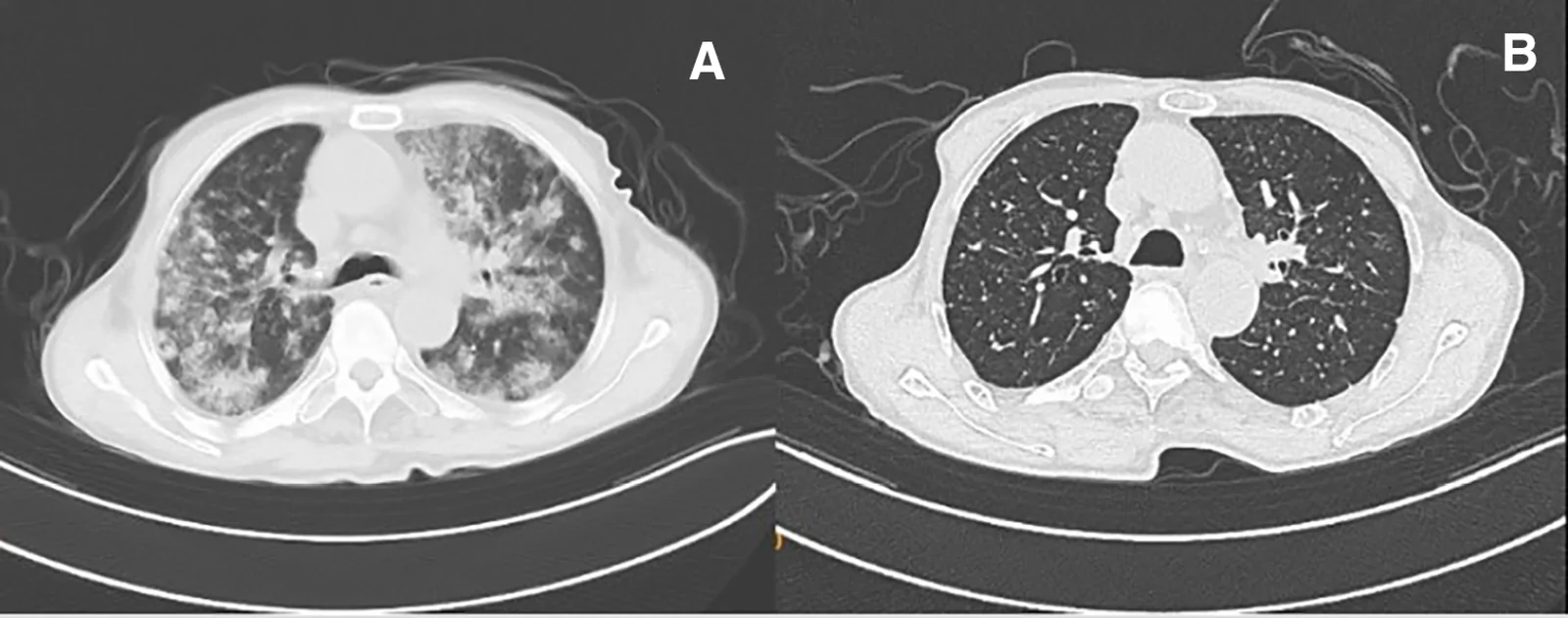
Serial chest CT. (A) June 2024: Right upper lobe tuberculous cavity with bilateral pulmonary infiltrates and effusions. (B) July 2025: Complete lesion absorption with fibrous scars after 12-month antituberculosis therapy. CT = computed tomography.

### 2.2. Anti-infection and supportive care

A standard 2HREZ/4HR antituberculosis regimen was implemented. Initial cefuroxime and piperacillin–tazobactam were replaced with moxifloxacin due to a poor therapeutic response.

Obvious remission of symptoms and inflammatory markers was observed after 2 weeks of standardized treatment. The patient completed a full 12-month antituberculosis course. July 2025 chest tomography exhibited nearly complete absorption of tuberculous lesions with residual fibrous scars, signifying clinical cure (Fig. 2B).

Longitudinal Immune Function Surveillance (3-year follow-up: June 2023–June 2026).

Peripheral lymphocyte subgroups and inflammatory cytokines were dynamically monitored over 3 years. At the initial August 2023 admission, total T, CD4^+^, and CD8^+^ lymphocyte counts were below normal limits, accompanied by elevated interleukin-4 (IL-4), IL-6, and interferon-gamma (IFN-γ), indicating baseline cellular immunodeficiency and persistent inflammatory activation. Two months after steroid initiation (October 2023), serum IgG4 dropped moderately while proinflammatory cytokines, especially IFN-γ, remained elevated. When dual infectious complications occurred in June 2024, peripheral T and B cell counts reached their nadir, matching the peak hs-CRP level of 162.99 mg/L. Even after cyclophosphamide withdrawal and complete infection remission in July 2025, T cell subsets failed to recover fully, which confirms permanent cellular immune impairment.

Serological data revealed divergent biomarker trends (Fig. 3): paraprotein maintained a stable concentration of 2.2 to 2.3 g/L throughout the whole observation period without signs of myelomatous transformation, whereas serum IgG4 rebounded to 4326 μg/mL during steroid tapering, suggesting mild IgG4-RD reactivation. Notably, hs-CRP only elevated during infectious or disease flare episodes, with no parallel fluctuation of paraprotein, which verifies independent disease progression of IgG4-RD and MGUS. Serial imaging identified no active pancreatic or pulmonary lesions during follow-up.

**Figure 3.**
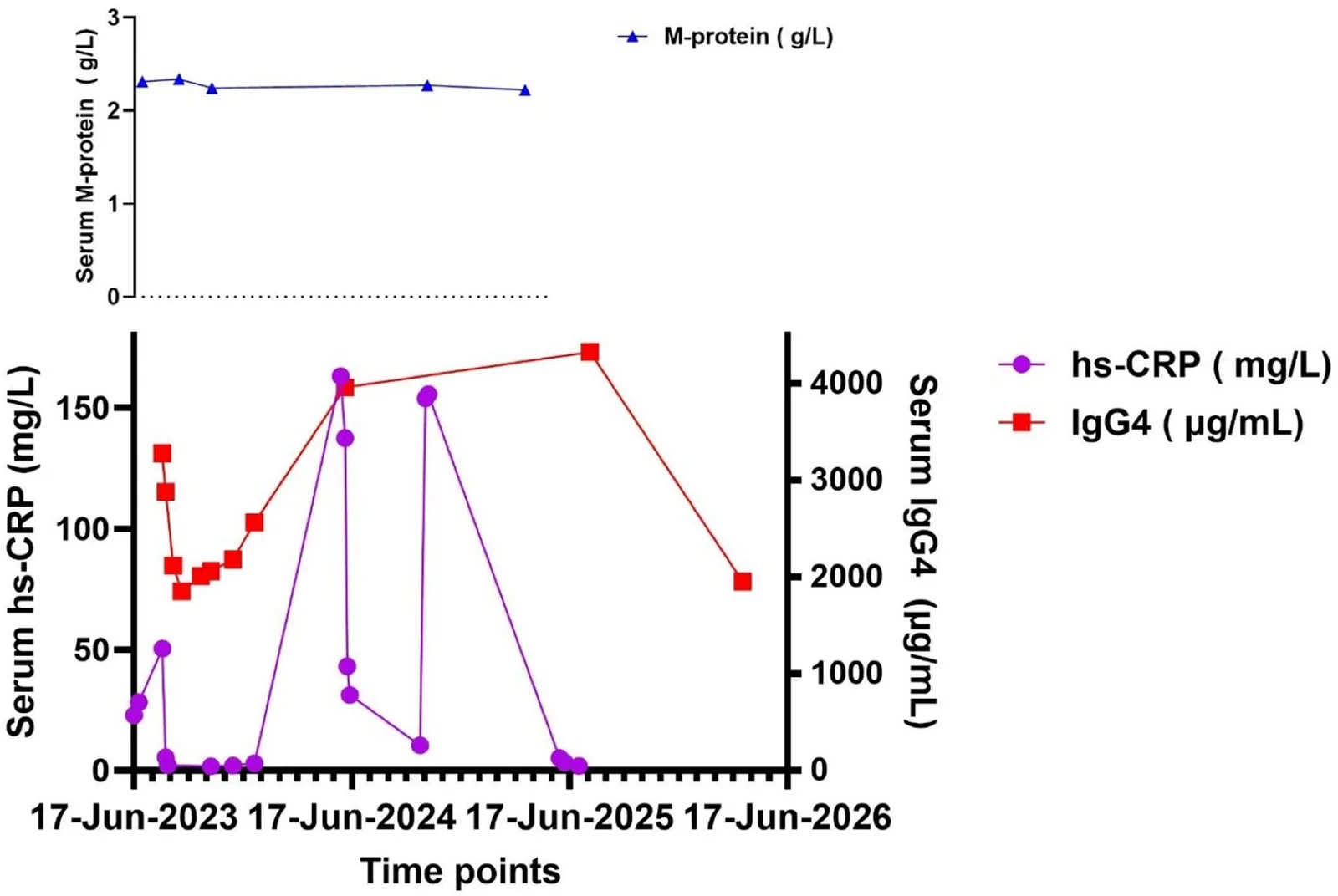
Longitudinal dynamic trends of serum hs-CRP, IgG4, and paraprotein over the 3-year follow-up period. The lower graph shows variations in hs-CRP (left vertical axis, mg/L) and serum IgG4 (right vertical axis, μg/mL). The upper inset enlarges the curve of paraprotein, which remained stable at 2.2 to 2.3 g/L with no obvious fluctuations during the entire observation course. hs-CRP = high-sensitivity C-reactive protein.

The patient’s comorbidities included post-PCI coronary artery disease, multiple cerebral infarcts, and severe osteoporosis. The long-term maintenance regimen adopted low-dose prednisone for IgG4-RD control plus ticagrelor and rosuvastatin for secondary cardiovascular prophylaxis, alongside symptomatic interventions for skeletal discomfort. Regular surveillance covering IgG4, paraprotein, free light chains, and chest CT was scheduled every 6 to 12 months to track disease activity and infectious risks.

## 3. Discussion

Published case series and isolated reports have separately documented IgG4-AIP complicated with MGUS or single mild infectious complications under immunosuppression; however, no prior work has recorded octogenarian patients experiencing concurrent *S. hominis* bacteremia and active tuberculosis accompanied by complete 36-month serial immune monitoring data.^[9]^ To place this rare clinical presentation into context, all peer-reviewed literature published between 2014 and 2026 focusing on overlapping IgG4-RD and MGUS was systematically reviewed. All retrieved reports adopted short follow-up windows (≤12 months) and only captured isolated mild infection events, without descriptions of dual life-threatening infectious episodes in patients over 80 years old. Aging-induced immunosenescence superimposed on dual plasma cell disorders creates an immune-vulnerable phenotype that cannot be predicted from younger patients with single immune disease. Serial lymphocyte and cytokine measurements obtained in this case provide unique longitudinal evidence of irreversible cellular immune dysfunction, a critical observation absent from all cross-sectional published data.

Advanced age inherently impairs T-cell generation and pathogen clearance capacity, elevating the reactivation risk of latent *Mycobacterium tuberculosis*. The patient developed 3 layers of superimposed immune impairment: chronic inflammatory injury driven by IgG4-RD, clonal plasma cell suppression from underlying MGUS, and profound lymphopenia induced by cyclophosphamide. These 3 pathogenic factors synergistically triggered simultaneous invasive staphylococcal bloodstream infection and tuberculosis reactivation. Throughout the 3-year surveillance window, peripheral T lymphocyte subsets maintained a sustained downward trend from baseline admission to the peak of dual infection; even after full infectious lesion resolution and cyclophosphamide discontinuation, T-cell counts never returned to normal levels. This persistent cellular immunodeficiency cannot be merely attributed to transient myelosuppressive drug toxicity. Instead, it originates from the stacked damage of age-related immune senescence, clonal plasma cell-mediated inhibitory signaling, and long-term overproduction of proinflammatory cytokines IL-4, IL-6, and IFN-γ. Sustained high cytokine concentrations continuously suppress naive T-cell proliferation and differentiation, forming a self-amplifying vicious cycle of progressive immune deterioration.

The proposed triple overlapping immune impairment model adequately explains the extremely rare co-occurrence of 2 distinct severe opportunistic infections in this elderly subject. Single-disease cohorts of isolated IgG4-AIP or senior MGUS carriers occasionally record mild solitary infections, yet concurrent bacteremia plus cavitary tuberculosis is almost never observed. MGUS clonal plasma cells inherently restrain physiological polyclonal immunoglobulin secretion and weaken innate antibacterial mucosal defense, while chronic IgG4-RD inflammation disrupts respiratory epithelial immune barriers. Once alkylating agents such as cyclophosphamide are added to control refractory pancreatic fibroinflammatory lesions, bone marrow lymphopoiesis is sharply inhibited, simultaneously crippling the body’s core defense lines against both mycobacteria and Gram-positive cocci. This triple-layer immune fragility cannot be anticipated by risk prediction tools designed for patients with only 1 plasma cell or autoimmune disorder.

Combined flow cytometry lymphocyte subset testing and serial cytokine quantification clearly delineate 3 interconnected immune injury pathways supporting the hypothesis:

1. Clonal plasma cell proliferation in MGUS suppresses physiological T-cell expansion, while persistent circulating immune complexes further impair baseline innate immune surveillance.
2. Chronic over-secretion of IL-4 and IL-6 under IgG4-RD depletes functional effector T lymphocytes, and short-term monotherapy with glucocorticoids fails to fully block sustained inflammatory signaling.
3. Combined glucocorticoid–cyclophosphamide regimens strongly inhibit bone marrow lymphopoiesis, with peripheral T-cell counts reaching the absolute nadir at the onset of dual infectious complications.

Three-year longitudinal serological trends illustrated in Figure 3 reveal independent activity trajectories of serum paraprotein and IgG4, filling an unaddressed knowledge gap regarding disease interaction in overlapping IgG4-AIP/MGUS populations. Serum M-protein remained stable at 2.2 to 2.3 g/L without end-organ damage throughout surveillance, while IgG4 concentrations fluctuated independently with steroid tapering and disease flares. This stable paraprotein level confirms MGUS acts as a concomitant premalignant plasma cell disorder rather than a secondary complication induced by IgG4-RD; the 2 entities share general immune dysregulation but possess distinct pathogenic driving pathways. Population-based studies report an annual 1% to 2% risk of MGUS progressing to multiple myeloma, which mandates lifelong regular paraprotein and free light chain surveillance for all affected patients.^[10,11]^

Large cohort data validate that MGUS doubles the cumulative risk of severe invasive bacterial and mycobacterial infections, consistent with the patient’s dual infectious phenotype.^[6,12,13]^ As confirmed by Cochrane systematic evidence, cyclophosphamide exerts potent inhibitory effects on both cellular and humoral immunity and significantly elevates tuberculosis and staphylococcal infection risk among IgG4-RD recipients.^[14,15]^ Mycophenolate mofetil was substituted for cyclophosphamide due to treatment-emergent liver injury; notably, irreversible T-cell suppression persisted long after infection cure and drug withdrawal. Glucocorticoids only transiently lower serum IgG4 without reversing baseline lymphopenia, creating an unavoidable therapeutic trade-off between controlling autoimmune inflammation and preserving antipathogen immunity. For this reason, cyclophosphamide should be minimized or avoided entirely in elderly patients with coexisting IgG4-RD and MGUS, and a stratified 3‑tier clinical management framework based on the 36‑month real‑world immune biomarker data from this case is constructed.

For patients aged ≥75 years with overlapping IgG4-AIP and MGUS, standardized pretreatment risk stratification is mandatory to reduce infectious adverse events. Baseline screening must include full lymphocyte subset quantification and IGRA latent tuberculosis testing to identify high-risk immune deficiency before initiating immunosuppression.^[16]^ For patients presenting pre-existing T-cell reduction at baseline, steroid-sparing agents with mild myelosuppression (mycophenolate mofetil) should be prioritized, and cyclophosphamide reserved only for refractory pancreatic lesions unresponsive to milder regimens. Stratified long-term surveillance schedules are recommended: high-risk patients with abnormal baseline immunity require serological, immune subset, and chest CT reevaluation every 6 months, while stable low-risk subjects may extend monitoring intervals to 12 months. Multidisciplinary collaboration across rheumatology, hematology, gastroenterology, and infectious diseases is essential to coordinate autoimmune disease control, infection prophylaxis, and hematological malignancy screening.

### 3.1. Limitations

This research carries inherent limitations stemming from its single-case design, which cannot generate quantitative population-level infection risk thresholds or stratified probability metrics for triple immune impairment progression. Large-scale multicenter longitudinal cohorts with standardized immune monitoring protocols are required to externally validate the triple immune injury hypothesis and refine the stepwise infection prevention algorithm proposed for elderly IgG4-AIP/MGUS patients.

## 4. Conclusions

Overlapping IgG4-AIP and IgGλ-type MGUS represents a rare combined autoimmune and plasma-cell disorder among elderly individuals. This 36-month longitudinal observation illustrates independent dynamics of serum IgG4 and paraprotein in the present patient. Triple superimposed immune impairment, consisting of IgG4-RD-driven chronic inflammation, MGUS-related clonal suppression, and drug-induced lymphopenia, substantially raises the risk of life-threatening dual opportunistic infection within this high-risk geriatric subgroup. Although patients with uncomplicated IgG4-AIP may develop immunosuppression-associated infections, they rarely exhibit this specific triple-component compounded immune impairment seen in the present case. Standardized pretreatment IGRA-based latent-tuberculosis and viral-hepatitis screening, individualized steroid-sparing therapy with restricted cyclophosphamide use, and semiannual immune-biomarker monitoring within a multidisciplinary framework are strongly recommended specifically for elderly patients with concomitant IgG4-AIP and MGUS. These clinical considerations are not generalizable to all patients with IgG4-AIP. Population-wide risk estimates cannot be derived from this single-case report. Multicenter prospective cohorts are required to validate the triple immune-impairment model and corresponding preventive protocols for this patient subgroup.

## Author contributions

**Data curation:** Wanman Li, Jiyuan Liang, Yao Xie.

**Investigation:** Wanman Li, Jiyuan Liang, Yao Xie.

**Formal analysis:** Tingting You.

**Methodology:** Tingting You.

**Visualization:** Tingting You.

**Conceptualization:** Li Chen, Jinhu Wei.

**Project administration:** Li Chen, Jinhu Wei.

**Supervision:** Li Chen, Jinhu Wei.

**Resources:** Jinhu Wei.

**Validation:** Jinhu Wei.

**Writing – original draft:** Wanman Li, Jiyuan Liang, Yao Xie.

**Writing – review & editing:** Li Chen, Jinhu Wei.
